# Editorial: The Role of Esophageal Manometry in Diagnosing Achalasia and Esophageal Motility Disorders: Challenges and Advances

**DOI:** 10.1002/jgh3.70093

**Published:** 2025-01-08

**Authors:** Kee Huat Chuah

**Affiliations:** ^1^ Gastroenterology and Hepatology Unit, Department of Medicine, Faculty of Medicine Universiti Malaya Kuala Lumpur Malaysia

**Keywords:** achalasia, asia, fundoplication, ineffective esophageal motility, manometry, POEM

Achalasia is a condition associated with significant morbidity and mortality. Among patients with achalasia, esophageal cancer, and pneumonia have been identified as carrying high mortality risks, with hazard ratios of 8.82 and 2.28, respectively [1]. Despite these risks, the diagnosis of achalasia is often overlooked. Although a German study demonstrated that the delay from symptom onset to diagnosis has shortened from 35 months to 20 months over 15 years, a diagnostic delay of nearly 2 years remains a cause for concern [2].

In this context, two large retrospective studies on consecutive patients undergoing esophageal manometry, conducted by Ghoshal et al. in India [3] and Abbass et al. in Pakistan [4], are particularly timely. The spectrum of manometric diagnoses varied across countries, but the frequency of achalasia was high: 56% in India and 55.9% in Pakistan. In Malaysia, 50.1% of patients with non‐obstructive dysphagia were diagnosed with achalasia (Table 1) [5]. Taken together, these findings suggest that achalasia is not uncommon in selected populations, particularly among patients presenting with dysphagia.

**TABLE 1:** Frequency of esophageal motility disorders on esophageal manometry.

	Malaysia [5]		India [3]		Pakistan [4]
Achalasia	50.1%		56.0%		
Achalasia type 1		27.5%		22.9%	62.0%
Achalasia type 2		62.5%		69.6%	30%
Achalasia type 3		10.0%		7.6%	8.0%
Variant Achalasia					
EGJOO	10%		3.3%		
IEM	12.5%		15.0%		
Absent contractility	7.5%		1.2%		
DES	7.5%		1.0%		
Hypercontractile esophagus	2.5%		0.1%		
Normal	10%		23.7%		

Abbreviations: DES, distal esophageal spasm; EGJOO, esophagogastric junction outlet obstruction; IEM, ineffective esophageal motility.

Interestingly, most patients with achalasia in India and Malaysia were classified as Type II, while those in Pakistan were predominantly Type I. Symptom duration before diagnosis averaged 18 months for patients under 60 years and 36 months for those over 60 years in India, whereas in Pakistan, it could extend as long as 8 years [3, 4, 5]. This prolonged duration in Pakistan may explain the higher prevalence of Type I achalasia, as Type I represents disease progression from Type II over time.

These findings highlight significant gaps in the timely diagnosis of achalasia. Greater education for clinicians to improve early detection and referral to tertiary centers is essential. Equally important is encouraging patients to seek medical consultation early. In line with these goals, the Malaysian Society of Gastroenterology and Hepatology and the Malaysian Upper Gastrointestinal Surgical Society have collaborated to highlight, recommend, and standardize the approach to managing patients with achalasia and esophagogastric junction outflow obstruction [6].

Treatment options resulting in good symptom improvement for achalasia are available. With the advent of newer treatment options, including peroral endoscopic myotomy (POEM), treatment outcomes have improved further. POEM has been found to be superior to pneumatic dilation for all types of achalasia and even better than Heller's myotomy for Type III achalasia [7].

Studies from India [3] and Malaysia also reported that ineffective esophageal motility (IEM) was the second most common diagnosis after achalasia (Table 1). IEM is commonly associated with GERD and was reported in up to 38% of patients with abnormal esophageal acid exposure [8]. However, IEM is often over diagnosed, particularly when using the Chicago Classification version 3 (CC v3), and may have limited clinical relevance in certain cases. In contrast, IEM diagnosed with the more stringent criteria of Chicago Classification version 4 (CC v4) has been reported to have a stronger association with pathological reflux [9]. The CC v4 protocol, which recommends conducting high‐resolution manometry (HRM) in both supine and upright positions, can help avoid underestimation of distal contractile integral (DCI), particularly if HRM is performed only in the upright position. Additionally, provocative tests, such as multiple rapid swallows, can evaluate peristaltic reserve; a lack of contraction reserve may support a diagnosis of IEM [10]. Although the latest gold‐standard protocols can be complex, they provide more convincing and accurate diagnoses, especially in inconclusive cases. Evaluating IEM and peristaltic reserve is particularly important for personalized treatment of GERD and anti‐reflux surgery. Tailored surgery, such as partial fundoplication for patients with GERD and esophageal dysmotility, rather than Nissen fundoplication, provides good symptom control while avoiding postsurgical dysphagia [11].

In conclusion, achalasia and other esophageal motility disorders are not uncommon in selected populations. HRM is an essential tool for evaluating patients presenting with non‐obstructive dysphagia and is a valuable diagnostic test for those with refractory GERD. Greater efforts are needed to train experts and expand the availability of HRM services globally to improve the timely diagnosis and management of these conditions.

## Conflicts of Interest

The author declares no conflicts of interest.
